# Counterfeit Healthcare Products: Nepal at a Vulnerable Position

**DOI:** 10.31729/jnma.7684

**Published:** 2022-12-31

**Authors:** Bijay Bhandari, Gaurav Rayamajhi

**Affiliations:** 1 Drug and Patient Safety Unit, Lumbini Provincial Hospital, Rupandehi, Nepal

**Keywords:** *counterfeit medicines*, *Nepal*, *patient safety*, *substandard medicines*

## Abstract

Nepal stands in a vulnerable position when it comes to counterfeit medicines as two of its major trade partners countries are leading producers of falsified healthcare products. The impacts that it can lead to are a lower standard of healthcare delivery to the public, increased cost of treatment, antibiotic resistance, and even fatality. The time of crisis as the Coronavirus disease 2019 pandemic is often seen as an opportunity to endorse substandard products in greater amounts. The people's doubt over the medicine is another issue generated by this malpractice that can lead to problems like vaccine hesitancy which can have scary outcomes so forth in a pandemic situation like this. Stronger national policies and vigilant authorities are prime to overcoming flourishing counterfeit culture as it is a peak time when we can not put public health at stake.

## INTRODUCTION

Nepal's production is mainly based on agricultural products and it has to rely on other countries for the majority of the industrially processed resources, be it because Nepal hasn't been able to thrive economically as an industrialised nation or because of the geographical challenge that it has been landlocked between these two giant countries. Moreover, countries from Asia top the list of the main producers of counterfeit pharmaceutical products and medicines traded worldwide.^1^ Undoubtedly Nepal can not be left uninfluenced in being such products marketed and consumed. World Health Organization (WHO) mentions, 25% of the medicines in lower and middle-income countries (LMICs) are counterfeit.^2^

## COUNTERFEIT MEDICINE IS A GLOBAL PROBLEM

Substandard and falsified medicines are an issue beyond borders. Food and Drug Administration (FDA) defines a counterfeit drug as "a drug which, or the container or labelling of which, without authorization, bears the trademark, trade name, or other identifying mark, imprint, or device, or any likeness thereof, of a drug manufacturer, processor, packer, or distributor other than the person or persons who manufactured, processed, packed, or distributed such drug and which thereby falsely purports or is represented to be the product of, or to have been packed or distributed by, such other drug manufacturer, processor, packer, or distributor".^3^ WHO Global Surveillance and Monitoring System for substandard and falsified medical products reports incidence of illness caused due to cough syrup in Paraguay and Pakistan which upon scrutinising found out that active ingredient 'Dextromethorphan' was imported from one of the South Asian countries which was further evaluated to find out that the chemical was levomethorphan instead of dextromethorphan which is five times stronger than morphine.^4^ Covering 10% of the global medicines market, counterfeit pharmaceuticals have a deep effect in LMICs where manufacture, importation, distribution, and sale are poorly regulated.^5^ A systematic review and meta-analysis reports overall prevalence of poor-quality medicines was 13.6% (95% CI, 11.0%-16.3%) in LMICs, with regional prevalence of 18.7% in Africa (95% CI, 12.9%-24.5%) and 13.7% in Asia (95% CI, 8.2%-19.1%). The most substandard or falsified drugs were antimalarials (19.1%) followed by antibiotics (12.4%).^6^ One hundred and twenty-three reports of substandard and falsified COVID-19 vaccines from 35 countries have been reported till May 2021.^7^ Southeast Asia is considered to be the hub for counterfeit pharmaceutical products. To state an example, the regulatory authority in this region had seized fake antibiotics (amoxicillin, ciprofloxacin, ofloxacin, cefixime) worth millions in 2016.^8^ In 2020, there were multiple reports of substandard medicines in the country from where Nepal imports different healthcare products.^1^

The compelling motivation for fraud producers is the humongous profit that counterfeit pharmaceutical business generates and digital access through online trade has made the business, even more, easier through unregulated websites.^9^ Only in the year 2016, the profit revenue garnered by the counterfeit medicines business is $4.4 billion.^1^ It has been found that producing a kilogram of fake viagra is more profitable than producing a heroine of the same volume.^10^ The International Criminal Police Organization (INTERPOL) has taken efforts to combat counterfeit and illicit goods among which pharmaceuticals are one they seized in INTERPOL-led operations worldwide.^1,11^

## DATA ON FALSIFIED MEDICINES IN NEPAL ARE SCARCE

"9 pc of drugs in the market are estimated to be substandard: DDA".^12^ An assessment of the quality of essential medicines in public health care facilities of Nepal in 2019, 37 batches of seven generics were found to be substandard.^13^ Another surveillance of quality medicine was done within Kathmandu valley in 2015, among 40 samples of eight different molecules of medicines investigated, 90% did not fulfil regulatory formalities in terms of labelling, and 42.5% did not meet pharmacopeial standards. Among them, 40% were domestic brands and 28% were imported.^14^ In 2018, INTERPOL-led "Operation Rainfall" saw Nepalese police seize 5,399 doses of opioid analgesics in a vehicle thought to have travelled from beyond borders.^11^ The drug procurement study in Nepal also clearly states that counterfeit products enter the system at the wholesaler and retailer level directly without prior formalities.^15^ Above studies are in favour that the counterfeit practice has its roots in Nepal.

WHO and INTERPOL both state that scammers have taken full advantage of the crisis of the ongoing COVID-19 pandemic to sell their fraudulent goods (hand sanitisers, face masks, personal protective equipment, and COVID-19-related medicines).^16^ However, this counterfeit trend in opportunistic times as a pandemic is not new and has been found to be practised even in Influenza and Ebola outbreaks.^17^ Such activities have direct ramifications on public confidence associated with medicinal products among which vaccine hesitancy is one of them, which can be a deliberate issue in an ongoing pandemic situation. Though there are meagre actions of drug recall as evident from the official website of the Department of Drug Administration (DDA), however, the drugs already reached the end users compromise the patients' safety.^18^

## TIME TO ACT

It is not just that these medicines will be hindering in achieving therapeutic outcomes but they can also lead to the development of drug resistance. Antibiotics were the most common among counterfeit medicines seized by the authority.^1^ The infective microorganisms when exposed to insufficient concentration of antibiotics, may develop resistance against those medicines. That is why antimicrobial resistance in LMICs is the burning issue and the major contributing factor is counterfeit or substandard medicines.

## WAY FORWARD

Weak national regulations, regulatory agencies, and investigational departments influenced by various means could be the reasons for increasing the counterfeit pharmaceutical business. Unless no reforms on regulations are done, thousands of people will continue to be affected due to counterfeit medicines. Authorities need to become vigilant and vigorous in managing the burgeoning counterfeit pharmaceutical business because the present public health condition is not up to the mark and the future can be even more fearful than it is at present.
